# Masculinity and Leadership Effectiveness (Self-)Perceptions: The Case of Lesbian Leaders

**DOI:** 10.3390/ijerph192417026

**Published:** 2022-12-18

**Authors:** Soraya Elizabeth Shamloo, Valeria De Cristofaro, Valerio Pellegrini, Marco Salvati

**Affiliations:** 1 Faculty of Medicine, University of Modena and Reggio Emilia, Viale A. Allegri 9, 42121 Reggio Emilia, Italy; 2 Department of Social and Developmental Psychology, Sapienza University of Rome, Via dei Marsi 78, 00185 Rome, Italy; 3 Department of Human Sciences, University of Verona, Lungadige Porta Vittoria, 17, 37129 Verona, Italy

**Keywords:** leadership, homosexuality, lesbian, effectiveness, masculinities

## Abstract

In line with the gay glass ceiling effect, sexual minorities are often target of discrimination within work-related contexts, thus potentially undermining their wellbeing at work. For gay men, discrimination may partially be attributed to gay men’s stereotypical feminine perception, which does not fit with the stereotypically masculine traits required for leadership positions. Yet, when considering lesbian women, the masculine stereotypical view associated with them may come to represent an advantage in work-related contexts, especially when compared to heterosexual women. In Study 1, N = 303 heterosexual participants rated a lesbian vs. a heterosexual woman as a job candidate on stereotypical gender (masculine vs. feminine) traits as well as leadership effectiveness. Results showed that being lesbian was associated with higher levels of masculinity (but not femininity), which in turn was related to high leadership effectiveness. In Study 2, N = 268 lesbian and heterosexual women rated themselves on the same measures. Results showed that both groups associated masculine traits with enhanced leadership effectiveness. These studies provide a better comprehension regarding how lesbian women may be perceived in work-related contexts and shed light on the role played by gender stereotypical perceptions in shaping both heterosexual and lesbian perceptions of leadership effectiveness.

## 1. Introduction

It is well known that sexual minorities are often the targets of discrimination [1,2]. Indeed, as part of a minority group, gay and lesbian individuals are not uncommonly discriminated against and, despite recent fights for equal rights worldwide, they still face social barriers [3]. One context in which sexual minorities face discrimination is the workplace [4,5]. Several studies have highlighted that living to your full potential at work and working in an inclusive and serene working environment are aspects positively associated with job satisfaction, which is a domain-specific facet of wellbeing, often referred to as ‘job-related’ wellbeing [6,7]. Few studies have focused on the wellbeing of sexual minorities in work-related contexts, showing that sexual minorities felt that their sexual identity could affect their career choices [8,9,10]. LGBTQ+ people with a positive self-image [11,12] might feel less constrained and pursue careers which they feel more drawn to and are of greater interest to them [13,14,15].

There are several ways in which minority–majority imbalance is perpetuated in the workplace. For example, some researchers have suggested that the modalities in which decision-making bodies are created may contribute to gender imbalance [16]. In addition, minorities such as LBGTQ+ individuals may experience resistance within the workplace, especially from those who undermine the importance of diversity at work. This is likely due to general confusion regarding why LGBTQ+ diversity is important within a work context [17], which may ultimately lead to a backlash—that form of resistance “to something that has gained, or is growing in popularity, importance, or power” [18] (p. 38). Resistance to change processes within work environments may also reveal itself through additional mechanisms (see [19] for a discussion), which include avoiding responsibility, blaming the disadvantaged, and assuming that there are more important priorities to deal with [20]. 

Importantly, the way in which individuals are perceived by society also plays a role in minorities being discriminated against in the workplace. As a case in point, the stereotypical belief that sees gay men as holding feminine, communal traits [21,22] and not embodying the masculine, agentic traits typically required for leadership positions [23] generally leads to discrimination towards this group. Similarly, the lack of masculine traits also represents one of the main reasons women are generally discriminated against when aiming for leadership positions [24]. Indeed, women are typically described as possessing stereotypically feminine, communal characteristics, which do not overlap with the masculine characteristics often associated with managerial positions. This likely prevents women and gay men from accessing leadership positions [25,26,27], in line with the so-called glass ceiling effect [28,29,30]. 

What about women pertaining to a sexual minority group as homosexuals? As far as the workplace environment is concerned, lesbian women represent a minority group (i.e., a sexual minority) within a potential minority group (i.e., women). Indeed, several work environments are known to be male dominated. In this specific case, the aforementioned situation may be perceived as a “double jeopardy” or “intersectional invisibility”, which describes the cumulative disadvantage experienced by people with multiple intersecting identities [31,32], and it represents an important aspect to consider within work environments and specifically when considering leadership positions.

It may be reasonable to think that the multiple disadvantaged identities lesbian women carry with them may on the one hand hinder their work-related success when considering leadership positions [33]. On the other hand, research shows that lesbian women are usually described as having stereotypically masculine, agentic traits [22,34,35], which partially overlap with the general masculine view associated with leadership positions. Based on these findings, one may put forward that this stereotypical view may offer lesbian women a chance of being perceived as more effective in leadership positions compared to heterosexual women. This will be investigated in the present research.

Indeed, while several studies so far have investigated how gay men are perceived in the workplace and specifically in leadership positions [27,36,37,38], less attention has been devoted to understanding whether lesbian women are perceived as efficient in leadership positions (but see [33,39]), as well as to the study of lesbians’ own perceptions of leadership effectiveness [40]. Focusing on this line of research is of extreme importance, since it would help better clarify the peculiar intersection between gender and sexual orientation that is present in the case of lesbian women. Indeed, especially as far as the workplace is concerned, lesbian women represent a discriminated minority group within another possible minority group and should thus be better investigated. 

To reach this aim, we specifically focused on how the attribution of masculine (e.g., competence, dominance) and feminine (e.g., affection, supportiveness) traits play a role in shaping heterosexual individuals’ perceptions of lesbian women’s leadership effectiveness (Study 1), as well as on lesbian and heterosexual women’s self-perception of leadership effectiveness (Study 2). 

### 1.1. Gender Stereotypes in the Workplace: The Case of Lesbian Women

When referring to men and women, society usually promotes specific gender-stereotypical perceptions which indicate how individuals should think and behave. Thus, stereotypical expectations define individuals by shaping the way people describe men and women [41]. Specifically, men are usually seen as more agentic (masculine) while women, on the contrary, are usually perceived as having communal (feminine) traits [42,43,44]. The term agency is usually conceptualized as including specific characteristics, such as achievement-orientation (e.g., competence), inclination to take charge (e.g., dominance), inclination to autonomy (e.g., independence) and rationality (e.g., objectivity). Differing from agency, communality entails concern for others (e.g., caring), affiliative tendencies (e.g., warmth), respect, and emotional sensitivity [45]. Importantly, these two components (i.e., agency and communality) are not mutually exclusive, given that high levels of one component do not necessarily mean low levels of the other component [46].

The difference between the general stereotypical beliefs held by society about men and women carries important negative consequences for the latter group when considering work environments and specifically leadership positions. According to the lack-of-fit model [47], this is mainly because leadership is often associated with masculinity and agency [23,48], characteristics which are typically thought to be lacking in women. Consequentially, women are often considered less able to hold managerial positions [49] compared to men, specifically because they are stereotyped as less agentic [25,26].

Back in the late 1980s, researchers started to question this model when considering sexual minorities, suggesting that this view may not hold for gay men and lesbian women. According to the “Gender Inversion Theory” [21,22], gay and lesbian individuals would be perceived as more similar to heterosexual individuals of the opposite gender; that is, lesbian women should be seen as more masculine, and gay men as more feminine. Indeed, research has shown that women who hold more masculine (i.e., agentic) traits are usually perceived to be lesbian, while males who have more feminine (i.e., communal) traits are likely to be perceived as gay [50,51]. In line with this reasoning, and with regard to work environments, lesbian female managers may be thought to partially hold similar traits to heterosexual male managers [23,48]. 

Yet, while several studies to date have investigated the perceptions of gay males in leadership positions, less is known about how lesbians are perceived in work environments, especially in leadership positions. Studies focusing on job competence suggest an existing advantage for lesbian women compared to heterosexual women [39], and similar results were also found when focusing on job hireability (e.g., [52,53,54,55]). Other studies specifically focusing on leadership positions have suggested the opposite [33,56]. These studies have mainly relied on voice cues associated with a lesbian vs. heterosexual potential job candidate and showed that heterosexual participants rated lesbian candidates as less adequate for leadership positions compared to their heterosexual counterparts. Yet, these studies specifically focused on voice as a cue to sexual orientation, which is known to be difficult to identify, rather than accurate detection of speakers’ sexual orientation. Indeed, individuals are much more accurate at identifying the speakers’ sexual orientation when heterosexual compared to lesbian voices are presented, thus indicating that several participants may not have been fully aware of the candidate’s sexual orientation. Nevertheless, these studies are extremely relevant as they indicate that voice indeed conveys meaning and guides first impressions. In addition, given that sexual orientation is not openly stated in work contexts, focusing on voice evaluation is extremely important as it may resemble real-life situations which individuals likely face when encountering (e.g., by phone) sexual minorities. In sum, these studies provide an insight into how such voice cues related to sexual orientation are perceived by society, and how these shape work-related decisions in terms of hireability and job suitability.

In sum, given the mixed findings, further studies are needed to better comprehend how lesbian women are perceived in work positions such as leadership positions. This research might contribute to filling this gap. 

### 1.2. The Present Research

The main aim of the present set of studies was to investigate perceptions of lesbians’ leadership effectiveness by heterosexual individuals (Study 1), as well as lesbian women’s self-perception of leadership effectiveness (Study 2). Previous studies show that successful leaders are perceived to hold stereotypically masculine (vs. feminine) traits [23,33,36,37,38,48] and that, in general, lesbian women are stereotypically perceived as more masculine compared to heterosexual women [21,22,57]. Putting these findings together, lesbian women, compared to heterosexual female individuals, might be perceived as more effective because they are generally perceived to be more masculine compared to heterosexual women [39,58]. In other words, since lesbian women are generally perceived and seen as more masculine compared to heterosexual women, this may in turn positively affect the perception of lesbian leaders as more effective (Study 1). We asked if this rationale could also be extended to lesbian women’s self-perceptions. In other words, we aimed to understand whether masculinity may also be associated with how lesbian women perceive themselves in terms of leadership effectiveness, and whether this relation differed between lesbian and heterosexual women (Study 2).

By specifically assessing both an outgroup’s (heterosexual) perception and lesbian women’s self-perception of leadership effectiveness, we aimed to provide two different points of view (i.e., an outgroup and the self) to reach a better understanding of the role of gender stereotypes in shaping leadership effectiveness perception.

## 2. Study 1

For Study 1, we hypothesized that both male and female heterosexual participants would evaluate lesbian women (compared to heterosexual women) as holding more stereotypically masculine traits [21,22,34,35,59] (Hypothesis 1a), which in turn would be associated with higher levels of leadership effectiveness [23,48] (Hypothesis 1b). In addition, we put forward that stereotypically masculine traits would mediate the relation between the leader’s sexual orientation and her leadership effectiveness (Hypothesis 1c).

In order to reach a better understanding of the role played by gender-stereotypical traits in shaping leadership effectiveness perception, in addition to masculinity, we also exploratively tested the role played by feminine traits. 

Male and female heterosexual participants were asked to read either a description of a 34-year-old heterosexual woman named Mary or a description of Mary, a 34-year-old lesbian woman. In other words, we manipulated Mary’s sexual orientation. Participants then evaluated Mary on a series of stereotypically masculine traits and stereotypically feminine traits, as well as Mary’s leadership effectiveness.

### 2.1. Method

#### 2.1.1. Participants

Three hundred and eight participants residing in the United Kingdom took part in the study. Sample size was established through a power analysis designed for models including two parallel mediators. The analysis was performed with an R application entailing a Monte Carlo simulation approach [60]. We estimated statistical power by setting a conventional power threshold of 0.80 and expected correlation equal to 0.25 among the predictor, mediators, and criterion. We also opted for a large total number of power analysis replications (5000) and wide Monte Carlo draws per replication (20,000). A minimum sample size of 250 participants yielded a statistical power of 0.83 (95% CI = 0.82, 0.84).

Five participants were eliminated because they had stated they were not heterosexual. The final sample comprised three hundred and three participants aged between 18 and 74 (*M_age_* = 35.23, *SD* = 11.72). Regarding self-reported gender, 151 participants were female, 152 were male. Participants were randomly assigned to one of two experimental conditions; namely, either to the lesbian woman leader condition (*n* = 152) or to the heterosexual woman leader condition (*n* = 151).

#### 2.1.2. Procedures

Participants were recruited via Prolific and received monetary compensation for taking part in the research. Before answering the questionnaire, potential respondents were asked to provide consent to participate in the study and to the aggregated use of their data. Following the demographic section of the questionnaire, participants were asked to read either a description of Mary, a 34-year-old heterosexual woman, or a description of another Mary, a 34-year-old lesbian woman. The sexual orientation of the candidate was clearly stated in her description as well as conveyed by giving participants information regarding the gender of the candidate’s partner. Participants were told that the description regarded Mary’s interests and hobbies and main personality characteristics (see Appendix A for Mary’s complete description). Thus, we manipulated Mary’s sexual orientation while all of the other characteristics of Mary’s description remained identical across the two conditions. They were also told that they were going to answer some questions about Mary on the following pages.

#### 2.1.3. Measures

*Gender*-*stereotypical traits*. Respondents were asked to rate Mary on stereotypically masculine and feminine traits. For this purpose, participants were provided with ten adjectives, five of which represented stereotypically masculine traits (i.e., dominant, competent, independent, assertive, self-confident) and five traits which were considered stereotypically feminine (i.e., supportive, affectionate, sensitive, warm, caring). Participants were asked to rate Mary on these traits on a scale ranging from 1 (not at all) to 7 (completely). These adjectives were selected from existing measures used in previous studies [27,59]. Cronbach’s alpha was 0.64 for the masculine traits and 0.84 for the feminine traits.

*Leadership Effectiveness*. The participants responded to the 10-item scale developed by Hais et al., [61] (see Appendix B), which was readapted for our study as already carried out in previous studies on gay leadership [27,36,38]. Participants were asked to rate the perceived effectiveness of Mary as a leader on a 7-point scale ranging from 1 (strongly disagree) to 7 (strongly agree): “Mary has the qualities for good leadership”, or “Mary would be an effective leader”. The scores for each item were averaged to form a composite score of leadership effectiveness, where higher scores indicated higher effectiveness. Cronbach’s alpha for this measure was 0.96.

In addition to these measures, participants were asked to provide additional information such as age, gender, and political orientation on a 7-point scale from 1 (extremely liberal) to 7 (extremely conservative).

## 3. Results

**Figure 1:**
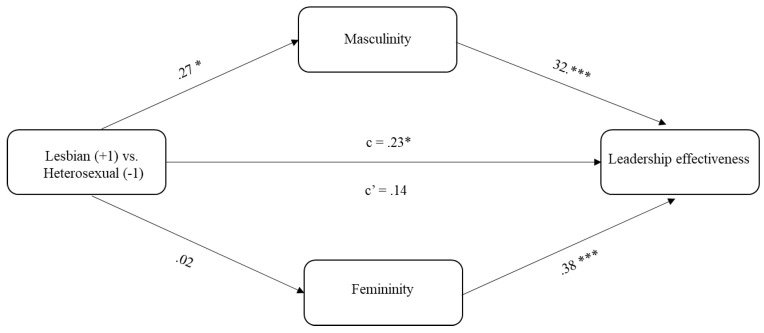
Proposed mediation model. *Note*. *** *p* < 0.001; * *p* < 0.05; Standardized coefficients are reported. c′ = direct effect of sexual orientation on leadership effectiveness; c = total effect of sexual orientation on leadership effectiveness.

Results of Study 1 are presented in Figure 1. Preliminary analysis confirmed that our manipulation was effective. Among respondents only *n* = 2, which is less than 0.7% of the sample, did not respond correctly when asked to indicate Mary’s sexual orientation, by indicating ‘heterosexual’ when Mary was presented as ‘lesbian’ or vice versa. 

Descriptive statistics were performed using the software SPSS, version 21 [62]. The mediation model was tested through the PROCESS macro [63] with analyses involving 5000 bootstrapping samples with 95% confidence intervals. Gender, age, and political orientation were controlled for. In the regression model, (Mary’s) sexual orientation (homosexual = +1; heterosexual = −1) was the predictor, stereotypically masculine and feminine traits were the parallel mediators, and perceived leadership effectiveness was the outcome variable. 

As predicted, and in line with Hypothesis 1a, Mary’s homosexual orientation was associated with a higher attribution of masculine traits (B = 0.17; SE = 0.07, *p* = 0.019, 95% CI: 0.0286, 0.3128). In addition, we found that sexual orientation was not associated with feminine traits (B = 0.01; SE = 0.08, *p* = 0.90, 95% CI: −0.1456, 0.1665).

In turn, as expected, masculine traits were associated with more leadership effectiveness (B = 0.45, SE = 0.07, *p* < 0.001; 95% CI: 0.3067, 0.5964), which was in line with Hypothesis 1b. Feminine traits were also associated with more perceived leadership effectiveness in our sample (B = 0.49; SE = 0.07, *p* < 0.001, 95% CI: 0.3549, 0.6187). 

As predicted, there was a significant indirect effect of sexual orientation on leadership effectiveness through more stereotypically masculine traits, confirming Hypothesis 1c (B = 0.08, SE(boot) = 0.04, 95% CI: 0.0126, 0.1606), while stereotypically feminine traits did not prove to be a significant mediator, (B = 0.01, SE(boot) = 0.04, 95% CI: −0.0718, 0.0843). The total effect of sexual orientation on leadership effectiveness was significant (B = 0.20, SE = 0.10, *p* = 0.042, 95% CI: 0.0077, 0.4012), while the direct effect did not prove to be significant, suggesting full mediation (B = 0.12, SE = 0.08, *p* = 0.13, 95% CI: −0.0343, 0.2789).

## 4. Study 2

In Study 1, we focused on male and female heterosexual individuals’ perceptions of lesbian (vs. female heterosexual) leader effectiveness and found that the relationship between sexual orientation and leadership effectiveness was mediated by masculine, agentic traits. Thus, heterosexual individuals tended to perceive lesbian individuals as more effective as a leader because of the stereotypically masculine traits attributed to them. Given the results found in Study 1 concerning the importance of stereotypically masculine characteristics in the relationship between sexual orientation and perception of effectiveness as a leader, in Study 2 we aimed to expand these findings by also investigating heterosexual and lesbian participants’ own perception of masculinity and leadership effectiveness. We were specifically interested in testing whether self-perception of stereotypically masculine traits was also associated with self-perceived leadership effectiveness. Additionally, we wondered whether this relation differed between lesbians and heterosexual women. 

We hypothesized that, in general, holding stereotypically masculine traits would also be associated with higher levels of leadership effectiveness when considering the self (Hypothesis 1), based on research showing how such traits are typically associated with leadership positions. In addition, we also investigated whether this relation could have been moderated by participants’ sexual orientation.

Indeed, as Study 1 indicated, society usually perceives lesbian women as more masculine [21,22,34,35] and being masculine leads to higher assessment of leadership effectiveness [23,48]. However, it is not known whether the stereotypical image of masculinity that is strongly associated with lesbians may be considered positively by this group. In other words, no research has yet investigated whether lesbian individuals would also consider their own masculinity traits to be a source of leadership effectiveness. Study 2 aimed to fill this gap. Specifically, given the lack of literature on this idea, we aimed to test two alternative hypotheses. On the one hand, we might expect both lesbian and heterosexual women to have internalized the stereotypes related to masculinity and its association with leadership effectiveness, applying it to themselves with no differences (Hypothesis 2a: absence of moderation). On the other hand, we could hypothesize that exposure to gender stereotypes that want lesbian women to be stereotypically masculine makes them more sensitive than straight women to associating their stereotypically masculine characteristics with leadership effectiveness. In this case, we would expect a stronger relationship between their own masculine characteristics and leadership effectiveness in lesbian women, compared to heterosexual women (Hypothesis 2b).

### 4.1. Method

#### 4.1.1. Participants

Two hundred and sixty-eight female participants residing in the United Kingdom took part in the online study. The sample size was established with an a priori power analysis by means of G*power software. The model tests an interaction between one dichotomous and one continuous variable, controlling for three covariates. Thus, we ran the analysis by setting a small f^2^ of 0.03, a conventional power threshold of 0.80 and an error probability of 0.05. With one tested predictor (i.e., the interaction) on a total of six (i.e., two main effects, the interaction, and three covariates), the analysis revealed a minimum sample size of 264 participants.

Respondents were aged between 18 and 69 (*M_age_* = 31.97, *SD* = 10.85). Regarding self-reported sexual orientation, 140 were heterosexual women, and 128 were lesbian women.

#### 4.1.2. Procedure

Participants were recruited via Prolific and received monetary compensation for taking part in the research. Since being female and heterosexual or homosexual was a necessary requirement for the study, only participants who had previously stated their heterosexual/homosexual orientation and female gender were invited to participate in the study. Before answering the questionnaire, potential respondents were asked to provide consent to participate in the study and to the aggregated use of their data. Following the demographic section, individuals answered questions about themselves which were presented as personality questions as well as questions about their perception of their own leadership effectiveness.

#### 4.1.3. Measures

*Gender stereotypical traits.* Respondents were asked to rate themselves by scoring same ten traits used in Study 1. As in Study 1, participants were provided with ten adjectives, five of which represented stereotypically masculine, agentic traits (e.g., dominant, competent) and five traits which were considered stereotypically feminine or communal (e.g., supportive, affectionate). Participants were asked to rate how suitable each of the characteristics were in describing their personality on a scale ranging from 1 (not at all) to 7 (completely). Cronbach’s alpha was 0.72 for the stereotypically masculine traits and 0.85 for the stereotypically feminine traits.

*Leadership Effectiveness.* The participants responded to the same 10-item scale used in Study 1 (adapted by Hais et al., [61] and already used in [27]). Participants were asked to rate their perceived effectiveness as a leader on a 7-point scale ranging from 1 (strongly disagree) to 7 (strongly agree). Example items include: “I have the qualities for being a good leader”, and “I would be an effective leader”. Scores were averaged to form a composite score of leadership effectiveness, where higher scores indicated higher effectiveness. Cronbach’s alpha for this measure was 0.95.

As in Study 1, in addition to the above-mentioned studies, participants were asked to provide additional information such as age, gender, and political orientation on a 7-point scale from 1 (extremely liberal) to 7 (extremely conservative).

## 5. Results

Analyses were performed using the software SPSS, version 21 [62]. To test whether perceiving oneself as masculine was associated with self-perceived leadership effectiveness, and whether this relation was moderated by sexual orientation, a simple moderation analysis was performed using the PROCESS macro [63]. Masculinity was entered as a predictor variable in the model, leadership effectiveness was the dependent variable, and sexual orientation (coded 0 = heterosexual and 1 = lesbian) was the moderator. We controlled for age, political orientation, and feminine traits. 

Results showed that the model was significant: *R*² = 0.44, *F*(6, 261) = 34.45, *p* < 0.001. Specifically, we found a significant and positive main effect of perception of masculinity on leadership self-effectiveness (*B* = 0.80, *SE* = 0.09, *p* < 0.001, 95% CI: 0.6305, 0.9662). Therefore, self-attribution of masculine traits was associated with higher levels of effectiveness as a leader for both lesbian and heterosexual participants. Importantly, the interaction between masculinity and sexual orientation did not prove to be significant, thus showing that sexual orientation did not moderate the relationship between masculinity and own leadership effectiveness (*B* = −0.06, *SE* = 0.12. *p* = 0.64, 95% CI −0.3075, 0.1902). This clarified that perceiving oneself as having more masculine traits was associated to more positive leadership self-perception regardless of one’s own sexual orientation. Further analyses not central to our hypotheses are presented as Supplementary Materials.

## 6. Discussion

Despite fights for equal rights worldwide, many groups, including sexual minorities, still face discrimination in the workplace [4,5]. These groups may experience resistance from majority groups through different mechanisms ([19] for a discussion), including avoiding responsibility, blaming the disadvantaged, and assuming that more important priorities should gain attention [20]. Importantly, the way in which individuals are viewed by society, in terms of gender-stereotypical traits [23,33,36,37,38,48] may also play a role in perpetrating discrimination within working environments. The present research was intended to address if and how gender stereotypes are associated with efficacy perceptions regarding lesbian individuals as leaders by the heterosexual majority, as well lesbian women’s self-perceptions as leaders. Specifically, the present research aimed at expanding the breadth of studies on leadership effectiveness by specifically considering lesbian leaders. To achieve this aim, two studies were carried out with the specific aim of better understanding what leads lesbian women to be perceived as effective in leadership positions by heterosexual individuals (Study 1), as well as lesbians’ self-perception of leadership effectiveness (Study 2).

In general, and in line with the “Gender Inversion Theory” [21], we hypothesized that lesbian women would tend to be regarded as effective because they are believed to possess stereotypically masculine traits to a higher degree than heterosexual women. Study 1 supported our hypothesis. Lesbian women tended to be considered as more stereotypically masculine compared to heterosexual women, which in turn was associated with more positive evaluations of lesbian leaders in terms of effectiveness. These results support previous studies showing how lesbian women are perceived as more masculine compared to heterosexual women [21,22,34,35] as well as research suggesting that masculine traits are associated with leadership effectiveness [23,48]. In sum, our results hint at the fact that lesbian leaders may indeed be regarded as more effective compared to heterosexual women because they are likely considered to be in line with the prototypical masculine, agentic leader [39,58]. 

Additional exploratory analyses of Study 1 also showed that stereotypical feminine traits were also associated with leadership effectiveness. This may suggest that in order to be effective, female candidates should also possess gender conforming traits. In sum, results suggest that the concept of a good leader does not only stem from regarding lesbian women as holding masculine traits but also from them having feminine traits, thus by also conforming to gender-stereotypical roles. This is in line with previous studies suggesting that to be perceived as a good leader, women should not undermine their feminine, communal aspects and thus not break gender roles [52,64]. For example, Niedlich and colleagues [52] showed that lesbian women, compared to heterosexual women, tend to be perceived as more competent in a job interview when they display behavior that follows gender roles (i.e., behavior typically associated with women). Niedlich’s results demonstrate the possibility of a lesbian advantage specifically when a cue regarding a traditional female gender role is presented prior to the candidate’s evaluation. Our results are also in line with studies showing how lesbian female managers are seen in ways consistent with heterosexual men, but also have something in common with heterosexual women [23], and thus that having communal traits rather than only masculine, agentic traits is important for leadership positions. 

Altogether, the results of Study 1 agree with other studies suggesting the possible advantage held by lesbians compared to heterosexual women in job contexts. However, our results should be taken with caution since studies have also suggested that this advantage may depend on additional factors which could interact with sexual orientation, such as the applicant’s gender role conformity (i.e., feminine behavior) or the gender type of jobs—that is, the extent to which a job is associated with stereotypically masculine or feminine traits [52,53,58] in line with the lack-of-fit model [47].

What about lesbian and heterosexual women’s self-perception? Our study also explored self-perception and its relationship with leadership effectiveness. Indeed, by also taking into consideration self-perception of leadership effectiveness, we addressed our aim of extending knowledge regarding lesbian leadership effectiveness. Research shows that in organizational contexts different identities often merge (e.g., lesbian and leader) into a sense of being and are both salient for individuals [65]. These results clarify some of the variables associated to a positive (self-)perception as a leader. Specifically, this study helped to understand whether stereotypical gender traits would be differently perceived, as regard to their importance for leadership effectiveness, by heterosexual and lesbian individuals. Study 2 showed that the relationship between self-ratings regarding stereotypical gender traits and leadership effectiveness did not differ between heterosexual and lesbian women. Specifically, the more stereotypically masculine lesbian and heterosexual women rated themselves, the more effective as leaders they perceived themselves to be. Although not central to our hypothesis, Study 2 also provided evidence for the importance of stereotypically feminine traits for leadership effectiveness, which was in line with Study 1. We found that for both heterosexual and lesbian women, there was a significant relationship between stereotypically feminine traits and leadership effectiveness, once again providing evidence for the fact that to be perceived (Study 1) as well as to perceive oneself (Study 2) as effective, lesbian women should still retain some feminine traits. 

### Limitation and Future Research Directions 

This study is not free from limitations. First, we did not consider different job descriptions in Study 1. In other words, we asked participants to rate leadership effectiveness in general, without focusing on a specific stereotypically masculine vs. stereotypically feminine job. Masculine and feminine traits may be perceived differently according to the female- or male-dominated job context. Thus, considering stereotypically masculine vs. feminine jobs could help clarify whether lesbian leaders compared to heterosexual candidates may generally be perceived as more effective as a leader in certain job areas compared to others. Thus, future studies might consider this situational factor too.

Second, the sample from Study 1 only included heterosexual individuals, yet it could be interesting to investigate lesbian individuals’ perception of their own ingroup, both in terms of stereotypical traits and leadership effectiveness. Thus, future studies should also explore the ingroup perspective to develop a clear picture of the population’s perspective on lesbian leadership effectiveness. 

Third, our study included personal information about the candidate (i.e., sexual orientation), which is not usually disclosed in hiring situations. Thus, future studies should also rely on other cues which may hint to the sexual orientation of the candidate without directly disclosing it, as other studies (e.g., [33]) have started to do (e.g., voice but also visual recognition which may together enhance the possibility of correctly identifying the sexual orientation of the candidate). 

Another point which needs further investigation regards whether lesbian women’s internalized sexual stigma could also play a role in participants’ self-perception of effectiveness as a leader. Indeed, previous studies on gay men have shown that individuals with high levels of internalized stigma report less self-perceived effectiveness as leaders [27]. Similar results may be found among lesbian women, yet no study has investigated this aspect so far. Future studies should fill this gap. 

Last but not least, a further important direction of research focusing on sexual minority members’ leadership might be a better inclusion of all the people that characterize the LGBTQ+ acronym. Indeed, social psychological research which addresses LGBTQ+ issues often focuses on gay men, leaving out not only lesbian women, but especially bisexual and trans people [2]. This is often due to difficulty reach these very specific targets of population, leading them to be invisible and ignored.

## 7. Conclusions

Further research on LGBTQ+ leadership is recommended as it has several research and applicative implications. Studies like this can provide novel insights about the key role of gender stereotypes in organizational contexts, where leadership characteristics are determinant in the hiring processes for managerial positions. Gender and sexual minority individuals who internalize such stereotypes by associating leadership with masculine characteristics might avoid applying for such positions, with the consequence of reducing their career expectations, while strengthening and perpetuating the gay glass ceiling effect. Being aware of these stereotypes, for instance by introducing diversity management programs in companies and organizations, could represent a useful strategy to contrast prejudice, making access to leadership positions within reach for all people who aim for them. However, according to Hill [18], in order to be effective, diversity management programs should consider different aspects which should, among others, include (a) defining diversity by considering individuals of all demographic groups; and (b) moving from policies focused on tolerance to policies centered on celebration of all people. This aspect is crucial since tolerance may convey the information that some individuals indeed have the authority not to tolerate ([66] cited in [18]). In addition, focusing on tolerance may somehow place the “dominant group at the center, and “Others” on the margins” [18] (p. 48). Hill also stresses the importance of relying on behaviors, communications, events, tasks, and organizational recreational opportunities which include every single employee. In this way, sexual minorities would feel free to live to their own full potential at work and pursue the careers to which they aspire, contributing to their job satisfaction and wellbeing. 

## Figures and Tables

**Figure A1:**
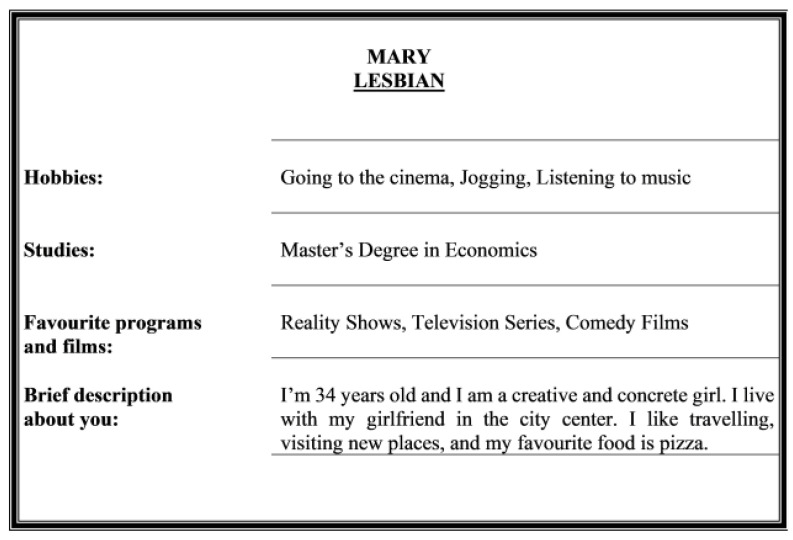
Lesbian condition.

**Figure A2:**
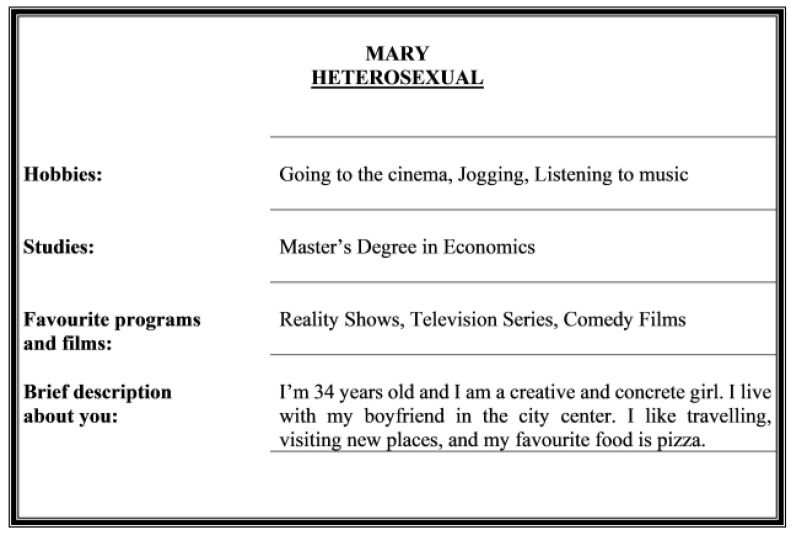
Heterosexual condition.

## Data Availability

The data that support the findings of this study are available from the corresponding author, upon reasonable request.

## Supplementary Materials

The following are available online at https://www.mdpi.com/article/10.3390/ijerph192417026/s1.

## Appendix A

Mary’s description.

## Appendix B

The following items were used to assess leadership effectiveness in Study 1 (adapted from [61] Hais et al., 1997). Items used in Study 2 were identical but asked participants to rate themselves.

(1) Mary has the qualities for being a good leader
(2) Mary’s image matches with the image of a good leader
(3) Mary would behave as a leader should
(4) Mary would be an effective leader
(5) I like Mary as a leader
(6) Overall, Mary would be a good leader
(7) I support Mary as a leader
(8) I endorse Mary as a leader
(9) Other people would be willing to defer to Mary
(10) Other people would be influenced by Mary
