# Supplementary material for: Masculinity and Leadership Effectiveness (Self-)Perceptions: The Case of Lesbian Leaders
Source: Int J Environ Res Public Health. 2022 Dec 18;19(24):17026. doi: 10.3390/ijerph192417026 (PMC9779703; doi:10.3390/ijerph192417026)
Supplement: Supplementary file 1 [file ijerph-19-17026-s001.zip › ijerph-2005130-supplementary.pdf]

Supplementary Material—(Study 2)

In order to have a clear picture of the variables associated with leadership self-effectiveness and based on Study 1, which showed that holding feminine traits is associated with effectiveness, the same analysis but with feminine traits as the predictor was carried out. As in the previous model, we controlled for age, political orientation, and masculine traits. In this case the model was significant, $R^2 = 0.45$, $F(6, 261) = 34.97$, $p < 0.001$. Specifically, perception of feminine traits was related to self-perceived leadership effectiveness (B = 0.30, SE = 0.10, $p = 0.004$, 95% CI: 0.0973, 0.5016). The interaction between femininity and sexual orientation was not significant, thus showing that sexual orientation did not moderate the relationship between femininity and one's own leadership effectiveness (B = 0.19, SE = 0.14, $p = 0.16$, 95% CI −0.0760, 0.4591).
